# Correction: MiR-29b/Sp1/FUT4 axis modulates the malignancy of leukemia stem cells by regulating fucosylation via Wnt/β-catenin pathway in acute myeloid leukemia

**DOI:** 10.1186/s13046-023-02794-y

**Published:** 2023-08-17

**Authors:** Bing Liu, Hongye Ma, Qianqian Liu, Yang Xiao, Shimeng Pan, Huimin Zhou, Li Jia

**Affiliations:** 1 College of Laboratory Medicine, Dalian Medical University, 9 Lushunnan Road Xiduan, Dalian, 116044 Liaoning Province China; 2 Department of Clinical Laboratory, Beijing Hospital of Traditional Chinese Medicine Affiliated to Capital University of Medicine Sciences, Beijing, 100010 China; 3 Department of Microbiology, Dalian Medical University, Dalian, 116044 Liaoning Province China

**Correction:** ***J Exp Clin Cancer Res*** **38, 200 (2019)**

**https://doi.org/10.1186/s13046-019-1179-y**

Following publication of the original article [1], wrong image was used in Fig. 5, specifically:

- Fig. 5d—CyclinD1 gel blot

The correct Fig. 5 is given as below:

**Fig. 5:**
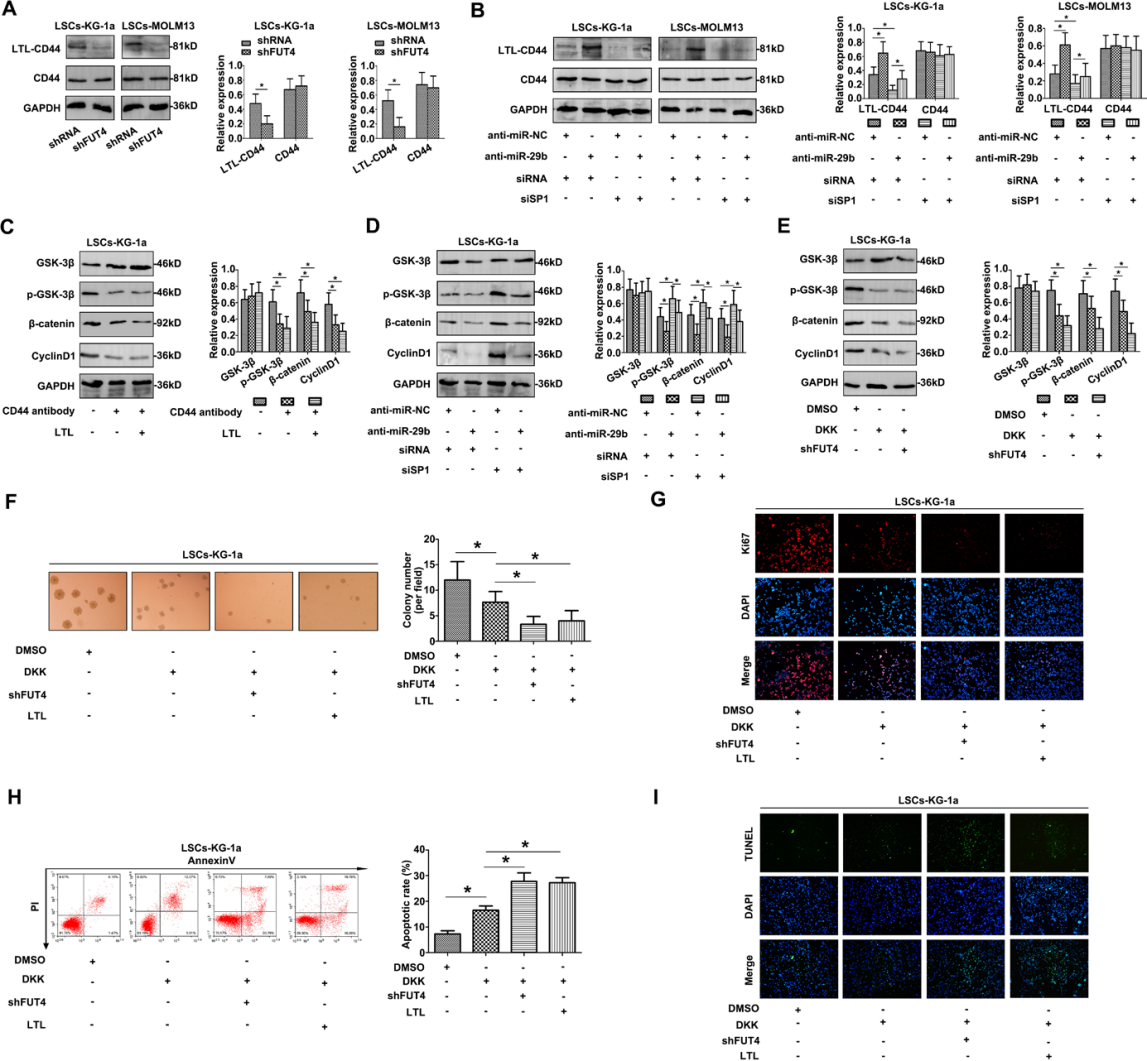
MiR-29b/Sp1/FUT4 crosstalk regulates CD44 fucosylation and activates Wnt/β-catenin pathway in CD34 + CD38- AML cell lines. **a** LTL-CD44 level was altered with mediation of FUT4, while total CD44 showed no changes. **b** Modulation of miR-29b and Sp1 caused the altered level of LTL-CD44, and showed no impacts on CD44 level. **c** With CD44 antibody and LTL treatment, the activity of Wnt/β-catenin pathway was inhibited in LSCs-KG-1a cells by western blot. **d** Co-transfection of anti-miR-29b and siSp1 also impacted the activation of the cascade by western blot. **e** Co-treatment of DKK and shFUT4 suppressed the pathway activity. **f** DKK and shFUT4 impacted the sphere formation ability of LSCs-KG-1a. LTL blocking assays also suppressed the proliferation. **g** Ki67 staining also indicated the attenuated proliferation of LSCs-KG-1a cells with the treatment DKK, shFUT4 or LTL blocking. **h** Apoptotic rates of LSCs-KG-1a were increased after DKK, shFUT4 treatment or LTL blocking by flow cytometry. **i** TUNEL staining confirmed the apoptotic occurrence. Data are the means ± SD of triplicate determinants (*P < 0.05)

The correction does not affect the overall result or conclusion of the article.
